# Immunological exhaustion: How to make a disparate concept operational?

**DOI:** 10.1371/journal.ppat.1009892

**Published:** 2021-09-23

**Authors:** Hannah Kaminski, Maël Lemoine, Thomas Pradeu

**Affiliations:** ImmunoConcept, CNRS & University of Bordeaux, Bordeaux, France; Universitat Zurich, SWITZERLAND

## Abstract

In this essay, we show that 3 distinct approaches to immunological exhaustion coexist and that they only partially overlap, generating potential misunderstandings. Exploring cases ranging from viral infections to cancer, we propose that it is crucial, for experimental and therapeutic purposes, to clarify these approaches and their interconnections so as to make the concept of exhaustion genuinely operational.

## Author summary

In this essay, we have written a critical review on immunological exhaustion. We believe that this widely used concept often remains in fact imprecise because there exist 3 different approaches to exhaustion, namely, in terms of dysfunction, cause, and marker, and those are not sufficiently well distinguished and articulated in most scientific papers. We also propose to talk about “exhaustion” for and only for the phenomena in which all 3 approaches are aligned. This can be called the “convergence strategy”: T cells would be described as “exhausted” if and only if they are simultaneously dysfunctional, express given markers such as programmed cell death protein 1 (PD-1), and induced by a specific cause such as chronicity. This strategy could be perfectly reasonable, and we see much value in adopting it, because it would force everyone to be more specific and rigorous when talking about exhaustion. Clarifying the characteristics of cells defined as “exhausted,” and using a convergent strategy to define T-cell exhaustion could have major experimental and clinical consequences, including for viral infections such as the Coronavirus Disease 2019 (COVID-19).

## Three approaches to exhaustion

Despite previous mentions to “exhaustion” in the immunological literature [1,2], the founding period for modern uses of the notion was the 1990s, during which exhaustion was defined both causally and functionally, focusing on specific CD8+ T cells during murine chronic lymphocytic choriomeningitis virus (LCMV) infection. “Exhausted” T cells are cells that, when exposed chronically to high quantities of antigen, are activated and proliferate, before becoming dysfunctional, i.e., unable to eliminate the virus. For Zinkernagel and colleagues [3], exhaustion is defined by the non-elimination of the virus, due to the peripheral deletion of all the virus-specific cytotoxic T cells, a concept validated a few years later [4]. For Ahmed and colleagues [5], exhausted cells are dysfunctional because their capacity to trigger an effector response against the virus is reduced, but these cells are maintained in the body. Overall, exhaustion has classically been defined both by dysfunction (these T cells fail to do what effector T cells are expected to do) and by a double cause (high viral load and chronicity). Following the work of Zinkernagel and Ahmed, the concept of exhaustion has most of the time been applied to CD8 T cells, although it is also sometimes attributed to other immune cells (especially CD4 T cells) (e.g., [6]). Both Zinkernagel’s and Ahmed’s groups worked on chronic LCMV infection in mice, which rapidly became, and still is today, the standard model for understanding exhaustion.

**Fig 1:**
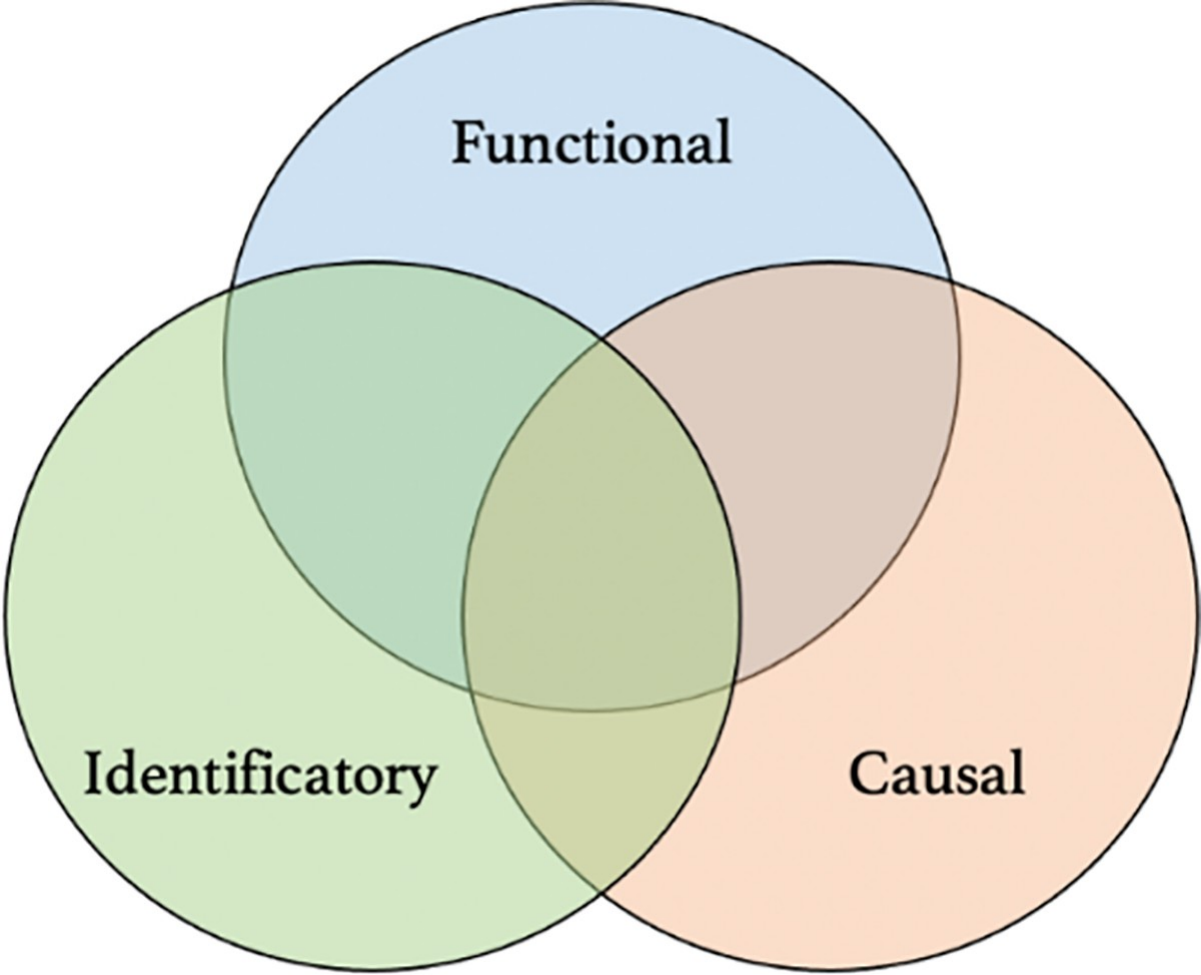
Three different approaches to immunological exhaustion. These approaches use different criteria and do not always overlap.

In subsequent research until present day, 3 approaches to immunological exhaustion have coexisted, with often unclear connections (**Fig 1**). The first approach primarily defines as exhausted the cells that present the same cellular dysfunction (typically, the absence of an expected effector response). The second approach primarily defines as exhausted the cells that are produced by a given cause (typically, but not necessarily, chronic exposure to an antigen). Finally, the third approach primarily defines as exhausted the cells that present the same molecular markers (typically, programmed cell death protein 1 [PD-1]). One difficulty is that authors tend to overlook specifying which approach they have in mind when they qualify cells as “exhausted.” A second even more serious difficulty is that authors often act as if these 3 approaches necessarily aligned (i.e., as if the 3 properties always occurred jointly), when in fact they don’t. As we will see, even the seemingly consensual view that antigen chronicity is the leading cause of exhaustion is questionable, including in the historically paradigmatic LCMV example. More recently, a subset of stem-like CD8+ T cells has been identified among exhausted T cells and presented as a much more rigorous characterization of exhaustion, but, as we will see, the nonoverlap problem also applies to this subset [7–10].

Below, we give several examples of nonoverlap, and we insist on the importance of both distinguishing and combining these 3 approaches to put together a precise and operational account of exhaustion.

**Fig 2:**
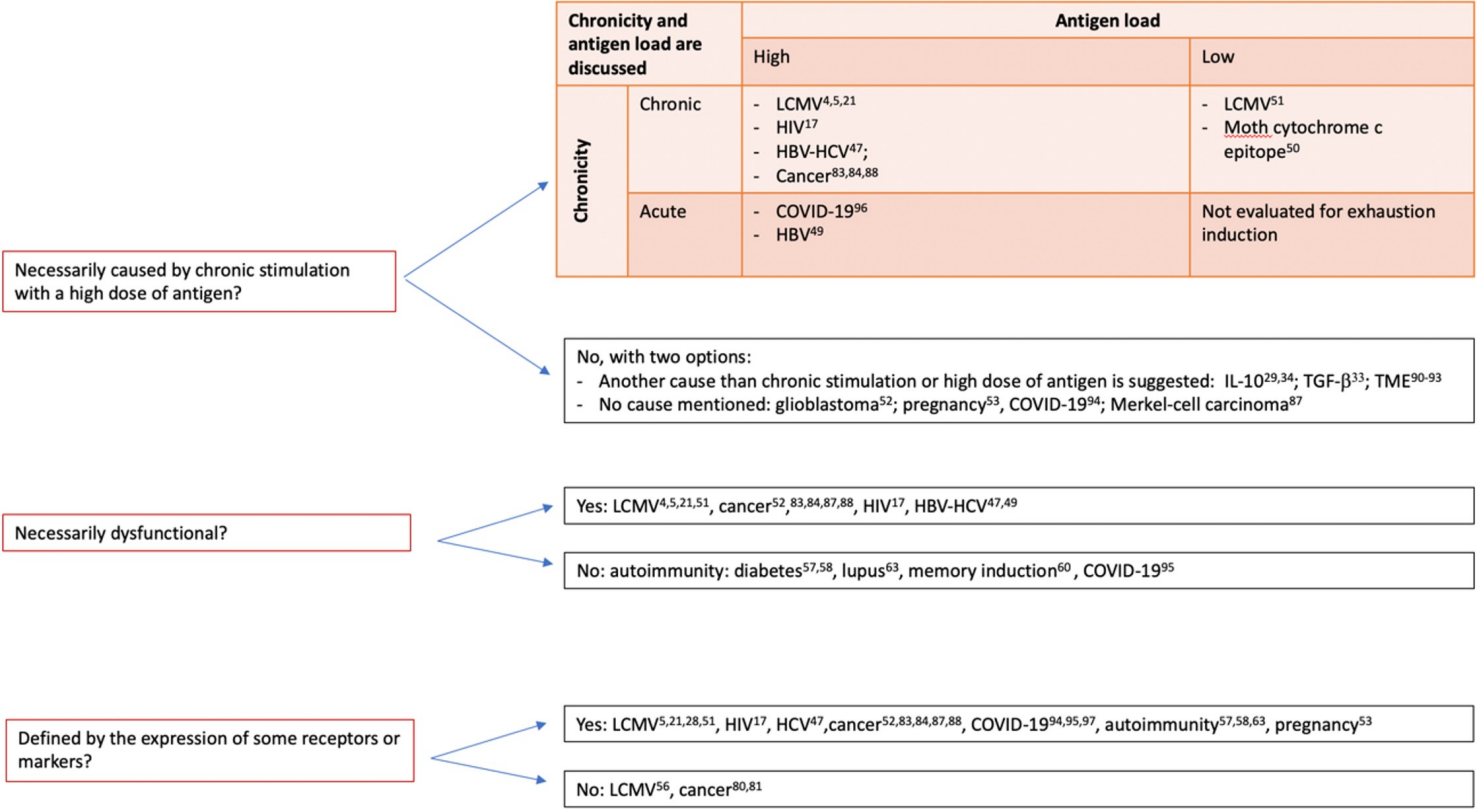
Key controversies about immunological “exhaustion”. COVID-19, Coronavirus Disease 2019; HBV, hepatitis B virus; HCV, hepatitis C virus; IL, interleukin; LCMV, lymphocytic choriomeningitis virus; TGF-β, transforming growth factor beta; Tumor Micro Environment (TME), xxx.

In our view, the lack of articulation between the 3 approaches distinguished above is the prime explanation for the current ambiguities and disagreements around the notion of exhaustion, as exemplified recently in the various and often competing positions expressed by 19 experts [11]. **Fig 2** sums up the main controversies about exhaustion.

Because we propose that it is essential for immunologists to distinguish the 3 approaches and make explicit in each experimental or therapeutic context which approach(es) they have in mind, we will try to present as precisely as possible these 3 approaches.

### The functional approach: Exhaustion is a cellular dysfunction

Originally, exhausted T cells were understood as dysfunctional. What is meant by “dysfunction” here is that some T cells, after activation and proliferation, do not fulfill the functions they are expected to perform as effector T cells—typically, they fail to eliminate infected cells and control the virus. As originally described, antigen-specific T cells become “dysfunctional” during the chronic phase of high viral load infections, with progressive loss of interleukin (IL)-2, then tumor necrosis factor alpha (TNFα), and, finally, interferon gamma (IFNγ). Authors have called these cells “dysfunctional” because they compare their functions in the context of a high-dose persistent chronic viral infection with the functions of the same cells in the context of acute viral infection when the virus is cleared [1].

In the original sense of “exhaustion” and especially for Zinkernagel’s team, the dysfunction that is characteristic of exhaustion can be defined independently of any cause or marker. Exhaustion is understood as the non-elimination of the virus due to the peripheral deletion of virus-specific cells. This constitutes a genuine functional characterization of exhaustion. Within this approach, saying that the cause of exhaustion is the exposure to high doses of a chronic antigen is therefore not circular: This cause is offered as the best possible explanation for an independently defined phenomenon.

The dysfunction associated with exhaustion has often been conceived in contrast to another dysfunctional state, namely “anergy.” The consensus is that anergy results from an absence of signal 2, and, therefore, a failure to become activated, while exhaustion occurs after T cells have been fully activated and have proliferated [12–14].

It is important to characterize as precisely as possible the nature of the dysfunction thought to be characteristic of exhaustion. For example, although this dysfunction has sometimes been conceived as involving an incapacity for maintenance and expansion [15–17], some T-cell populations (the stem-like CD8+ exhausted T-cell subset [18]) can persist and expand in chronic infections [13]. We now know that the exhaustion of such stem-like cells is, at least in some situations, reversible [12], contrasting with the long-held view that exhaustion would necessarily be irreversible [3,19–21]. The reversibility seems to concern especially T cell factor 1 (TCF1)+ cells. These PD-1^+^CD8^+^ T cells resemble stem cells during chronic LCMV infection, undergoing self-renewal and also differentiating into the terminally exhausted CD8^+^ T cells [9]. However, interferon regulatory factor 4 (IRF4) inhibition also seems able to induce TCF1 and leads to exhaustion reversibility [22].

“Exhausted” cells transferred into a naive host can reexpand and protect it against the same pathogenic challenge [23], suggesting that such cells are not irreversibly “dysfunctional,” and this restoration is now known to occur through the stem-like exhausted cells [9]. However, the dysfunction of stem-like cells concerns maintenance, expansion, and differentiation but not cytokine production (IFNγ) and cytotoxicity (granzyme B) since the terminally exhausted cells originating from the stem-like ones have the highest cytotoxic potential [9].

Is exhaustion always dysfunctional? There are important disagreements on this issue, some authors considering that exhaustion is by definition dysfunctional, while for others, exhaustion could be functional in some contexts [11,23,24]. One major limitation is that, in general, exhausted T cells are said to be “dysfunctional” with a unique and preexistent idea of “function” in mind—namely, the function to eliminate the virus. This expected and unfulfilled function remains in almost all cases the unique focus of the observer, which means that other active functions, such as immunoregulation or tissue repair, for example, are generally not explored. This makes it impossible to determine if, by becoming “exhausted,” cells lose some functions while simultaneously acquiring other functions. Major examples of contexts where so-called “exhausted” T cells could in fact play essential functional roles include the limitation of immunopathology in infection [25] and the regulation of autoimmunity [26].

### The causal approach: Exhaustion as resulting from specific causes

A second major approach is to say that exhausted cells are cells that are produced by a particular cause, typically antigenic chronicity and/or high antigen load. In the original LCMV model [3,5], both chronicity and antigen load were considered essential causes leading to exhaustion.

In subsequent work, antigenic chronicity remained an often mentioned typical cause leading to exhaustion [21,27]. Naturally, an important challenge when saying that exhaustion results from chronic exposure to an antigen is to determine what is meant by “chronic,” i.e., what the exact time course of exhaustion appearance is. Despite the general consensus that exhaustion is related to antigen chronicity, recent results have shown that this connection does not always hold. Although exhaustion has been classically observed during the chronic phase of uncleared viruses, it has been shown recently that, as early as 9 days postinfection of C13 LCMV, CD8 T cells lose their ability to make TNFα and IL-2 [28,29], which seems a very short time to refer to some type of “chronicity.” It has been suggested that thymocyte selection associated high mobility group box (Tox) determines early the T-cell exhaustion fate, but this has not been formally demonstrated yet [28]. A high dose of C-13 (10^6^), as opposed to a low dose of the same virus (10^2^), leads to exhausted cells by day 8 [9], suggesting that the antigenic load prevails over the chronicity of antigen exposure in T-cell exhaustion.

A second cause said to trigger exhaustion is indeed high antigen load. As previously said about the historical LCMV model, the claim that T cells were exhausted in C-13 by contrast to the Armstrong strain was based on the fact that the viral load was both higher and chronic [5,21]. The insistence on the role of a high antigen load also has roots in the concept of “immune paralysis.” This concept, widely used in the 1960s to 1970s [2,30,31], proposed that the immune system could become inoperant when confronted with high doses of antigen [21,28]. Later, some papers insisted on the crucial importance of high antigen load in the characterization of exhaustion, with or without an accompanying mention of chronicity [21,29,32]. A key challenge for this approach is to determine what quantity of antigen constitutes a “high dose” [21].

Importantly, other causes of exhaustion, beyond chronicity and antigen load, have been suggested, including contextual causes (**Fig 2**). One such additional cause is the cytokine environment, with a typical focus on IL-10 and TGF-β (which tend to favor exhaustion) [29,33,34] as well as IL-21 (which, in contrast, tends to inhibit exhaustion) [35]. Incidentally, the impact of the cytokine environment on the functionally defined exhausted phenotype of CD8 T cells demonstrates that these cells are not intrinsically “exhausted”; instead, exhaustion in the functional sense appears to be context dependent. Exhaustion is also influenced by causes at the cellular level, including both regulatory T cells [36,37] and myeloid-derived suppressor cells [38].

### The identificatory approach: Exhaustion as the cellular state associated with the expression of particular molecular markers

According to the third approach, exhaustion is the cell state associated with the expression of particular molecular markers—classically inhibitory receptors. Because there are many of them, only the most discussed in the scientific literature are mentioned here.

PD-1 has been the most extensively studied marker of exhaustion. It is considered as characterizing exhausted T cells in both chronic infection and cancer and associated with dysfunction in both cases [39,40]. In addition to PD-1, there are other receptors often considered as markers of exhaustion, often but not always in association with PD-1 (lymphocyte activation gene 3 [LAG3], T cell immunoglobulin and mucin domain-containing protein 3 [TIM3], T cell immunoreceptor with Ig and ITIM domains [TIGIT], cytotoxic T-lymphocyte antigen 4 [CTLA-4], etc.).

Some transcription factors have been interpreted as favoring exhaustion, for example, B lymphocyte–induced maturation protein 1 (Blimp-1), Eomesodermin (Eomes), Tox, and IRF4 (reviewed in [41]). During chronic infection, Eomes has been correlated with the “more terminal T_ex_ subset” [42]. Tox has been considered as a key transcription factor of the T-cell exhaustion program, since it was highly expressed in T cells during C-13 LCMV infection, whereas it was only transiently expressed at low levels during acute infection with Armstrong [28]. Moreover, T-cell exhaustion in cancer and chronic infection mainly relies on the presence of Tox, itself driven by chronic TCR stimulation and nuclear factor of activated T cells (NFAT) activation and associated with the expression of other transcription factors that are required for exhausted T cells (TCF1 and Eomes), as well as with inhibitory receptors and decreased function [28]. IRF4 contributes to inducing inhibitory receptors and leads to decreased functionality of CD8+ T cells during the chronic phase of LCMV infection and during cancer [22,43,44].

Finally, exhausted cells display metabolic changes such as inhibition of aerobic glycolysis due to glucose transport limitation and consumption by cancer cells, mitochondrial dysfunction, and oxygen deprivation [45], which, in turn, decreases cytokine production [46].

## The incomplete overlap between the 3 approaches to exhaustion

A common attitude is to assume that the 3 approaches to exhaustion generally align: Dysfunction would be produced by well-identified causes such as chronicity and/or high antigen load, and it would be associated with the expression of well-identified markers such as PD-1. In reality, though, these 3 approaches often fail to overlap. A lot of data suggests that exhaustion in the functional sense does not always causally result from antigen chronicity. Exhausted T cells have been described in chronic infection with LCMV, HIV [17], hepatitis B virus (HBV), and hepatitis C virus (HCV) [47], but not in cytomegalovirus (CMV), for example. Even if the 2 different kinetics of viral load have been mentioned as an explanation for the 2 distinct situations (exhaustion in the case of LCMV and inflation in the case of CMV), a better understanding of the cause involved is needed (CMV is thought to give series of low and short multiple replication periods contrasting with high chronic level of replication during LCMV, but studies on CMV suggest that the virus could still replicate actively in the tissues despite its absence in peripheral blood [48]). Moreover, the analysis of the profile evolution of CD8+ T cells in several infections shows that exhaustion in the sense of a dysfunctional phenotype occurs during the acute phase of C-13 LCMV infection, but not in other infections such as influenza, vesicular stomatitis virus (VSV) or *Listeria monocytogenes* [28], and HBV [49]. In a number of cases, antigen chronicity has been shown to be only partially related to exhaustion in a functional sense: Depending on the epitope, antigen load [27,50], and duration of infection, antigen-specific CD8 T cells responding to chronic antigen exposure may be fully functional, partially exhausted, fully exhausted, or physically deleted [21]. In other instances of exhaustion, we simply don’t know if antigenic chronicity plays a role or not. For example, in patients with non-small cell lung cancer, hepatocellular carcinoma, and glioblastoma [28,51], or during pregnancy [52], there is no kinetic approach that would establish whether the chronic exposure to tumor or fetal antigens played a role in the induction of T-infiltrating or peripheral blood lymphocyte exhaustion.

Another significant (and related) challenge is that exhaustion in the functional sense is not always caused by exposure to high doses of antigen. It has been recently shown that, in different infections, the adoption of an “exhausted” dysfunctional phenotype by antigen-specific CD8+ T cells occurred before the viral outcomes diverged, suggesting that viral load was not a primary driver of differential expression [28].

Regarding the markers used to define exhaustion, lack of overlap can also be observed. First, although PD-1 is the most often mentioned inhibitory receptor associated with exhausted T cells, it is certainly not a specific marker of exhaustion, as it is expressed after acute TCR activation [53]. The expression of PD-1 by non-exhausted cells is a widely recognized phenomenon [27]. Minimally, this forces us to recognize that PD-1 might be a necessary, although insufficient, marker of exhaustion and that exhaustion corresponds to a cluster of several markers rather than just 1 marker [54]. Saying that exhaustion is associated with a cluster of markers without being able to specify which is unsatisfactory. Moreover, PD-1 is not always required for the induction of exhaustion, and some features of exhaustion can even be more severe when PD-1 is absent [15]. Finally, the role of PD-1 has been highlighted in many other contexts than exhaustion, including autoimmunity [55], central and peripheric tolerance [56,57], acute infection for memory response [58], and balance between efficient anti-infectious defense and immunopathology [59]. In those contexts, PD-1 was not always associated with cellular dysfunction, and it was even sometimes associated with an increased memory response [57,58]. Thus, the function of PD-1 is interpreted differently depending on where and when it has been studied—sometimes as a sign of “exhaustion,” sometimes not. It seems that the most appropriate description would be that PD-1 is expressed in contexts of inhibition, rather than contexts of exhaustion per se, in so far as “exhaustion” is generally used when PD-1 is expressed in a context that is detrimental to the organism, but not when the context is beneficial (e.g., in peripheral tolerance during autoimmunity [60] or during T-cell memory formation [58]). Conversely, anti-PD1 therapy has been associated with the development of autoimmune diseases such as type 1–like diabetes [61].

Transcription factors also have ambivalent roles depending on the context. For example, Eomes has been associated with exhaustion during the chronic phase of infection, but it is up-regulated during acute infection, favoring effector molecule production (IFNγ), IL-15Rβ, and memory development [42,62]. Tox expression, highlighted as a key component of the exhaustion program, can in fact be already observed at day 4 of infection before antigen loads differ [28], which suggests that neither the chronicity nor the viral load are primary drivers of its expression. Moreover, the deletion of Tox restores the polyfunctional profile transiently but not in the long term, weakening the idea that this marker necessarily overlaps with dysfunction. Since the cause mentioned by the authors was chronic TCR stimulation, they hypothesized that the TOX program prevents overstimulation of T cells and immunopathology [39,63], which constitutes an alternative function rather than a dysfunction properly speaking. Consequently, the role of the differential expression of Tox in C13, Armstrong, and other microbial infections remains poorly understood.

IRF4 has been described both in LCMV infection and in allotransplantation, with a potential dual role. It shows a pro-exhaustion role during LCMV and cancer. In contrast, the deletion of IRF4 in CD4 T cells resulted in dysfunction and graft tolerance, which means that, when expressed, IRF4 represses PD-1 and Helios [64] and favors reactivity to the allograft.

Finally, the metabolic changes observed in exhausted T cells can also occur in the tumor microenvironment through causes distinct from antigen chronicity and high antigen load [65].

The upshot is that, despite the frequent tendency to consider the 3 approaches to immunological exhaustion to be aligned, it is often not the case. Some situations instantiate “exhaustion” from the functional viewpoint but are not associated with the classic molecular markers of exhaustion and/or the classic causes of exhaustion. This absence of convergence between the 3 approaches can be extremely problematic, as we can illustrate it now with the examples of cancer and Coronavirus Disease 2019 (COVID-19).

## Two illustrations of issues raised by incomplete overlap: Cancer and COVID-19

The fact that the 3 approaches to exhaustion do not always conceptually overlap can generate significant issues at the experimental and clinical levels.

The application of the notion of “exhausted T cells” to cancer is particularly telling. The aim of cancer therapies based on immune checkpoint inhibitors is to target dysfunctional T cells in cancer by reversing their state of tolerance to the tumor [66,67]. In recent years, many have connected the literature on immunotherapies with the preexisting literature on immunological exhaustion and claimed that targeting dysfunctional T cells in cancer amounted to targeting exhausted T cells [41, 68–70]. Importantly, most studies on T-cell exhaustion in cancer have been done in mice (e.g., [67,71]), but some have been done in humans (e.g., [72–74]).

**Fig 3:**
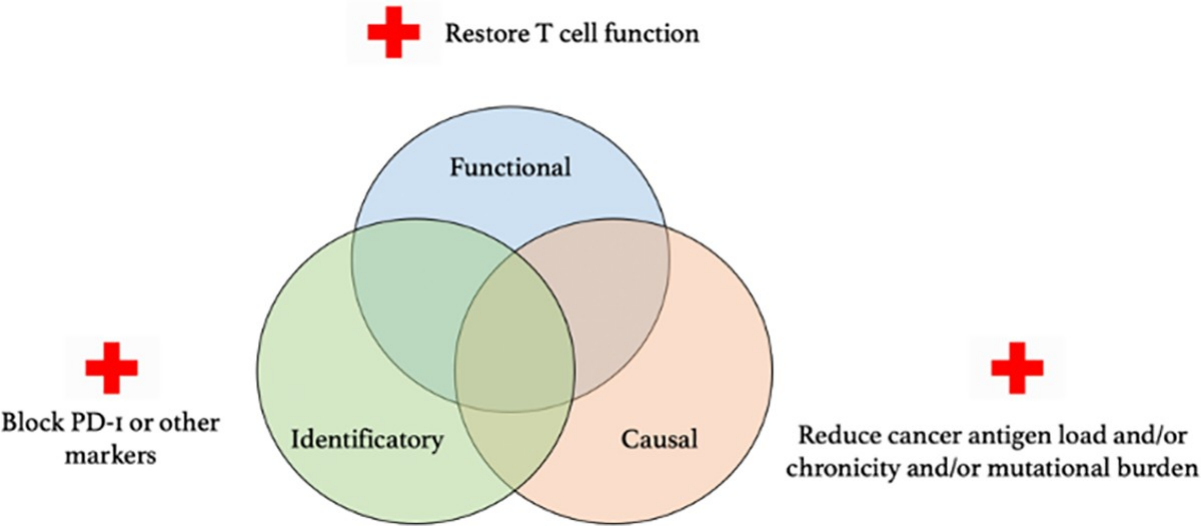
Targeting “exhaustion” in cancer therapies means different things depending on the approach to immunological exhaustion one adopts. PD-1, programmed cell death protein 1.

The claim that targeting dysfunctional T cells in cancer is equivalent to targeting exhausted T cells is, however, problematic for at least 2 fundamental reasons. First, for the clinician, targeting “exhausted T cells” will have a very different meaning depending on whether what is targeted is the dysfunction itself, the underlying causes, or the markers of exhaustion (**Fig 3**). Acting as if the 3 approaches always overlapped in cancer would be inappropriate and misleading, as (1) there are cases of cancer where some markers of exhaustion are dissociated from the causes of high antigen load and chronicity [75]; (2) dysfunction sometimes occurs without the expression of traditional markers of exhaustion [71]; and, conversely, (3) traditional markers of exhaustion are sometimes expressed in functional cells [76,77]. This confirms the importance of systematically specifying which meaning of “exhaustion” one has in mind when suggesting targeting exhaustion in a cancer setting.

Crucially, even when focusing on one specific and explicitly described approach to exhaustion, many important difficulties remain. This can be illustrated with the application to cancer of the causal approach of exhaustion. The main suggestion in papers linking cancer and exhaustion has been that the causes characterizing exhaustion in the viral context would also occur in cancer: The chronic exposure of T cells to a high load of tumor antigens would lead to their exhaustion, and, therefore, to the incapacity of these T cells to control tumor growth. This idea led to the hypothesis that poor immune responses to cancer are related to chronic exposure to high levels of tumor “neoantigens” [78,79] (typically seen as a consequence of a high rate of genetic mutations [80,81]). In clinical practice, the expectation has been that a high mutation load would lead to exhaustion, and, therefore, to good responses to anti-PD1 therapies. This is indeed what is seen in some cancer types [82]. For example, neoantigen burden in non-small cell lung cancer is directly correlated with the clinical response to anti-PD-1 therapy [83]. Yet, in a number of other cancer types, the correlation does not hold. Some cancers, such as the renal cell carcinoma, have a better response to anti-PD-1 therapy than the one that would be predicted by the tumor mutational burden. Others, such as colorectal cancer with mismatch repair proficiency, have a response that is worse than the one that would be predicted by the tumor mutational burden [82]. Overall, assuming an overlap between dysfunction on the one hand and chronicity and/or high antigen load on the other when predicting T-cell exhaustion and thus anti-PD1 response would lead to unsuitable therapeutic strategies.

Second, even in the limited number of cases in which there is a satisfactory overlap between the 3 approaches to immunological exhaustion (dysfunction, causes, and markers), focusing on exhaustion remains problematic because it constitutes at best a small subset of the many forms of immunological tolerance to the tumor. There are, in fact, many parameters that help explain the elimination or non-elimination of the tumor by T cells, and each of these parameters can be linked to biomarkers—as suggested by the concept of the “cancer immunogram” [84]. Furthermore, even the focus on CD8 and CD4 T cells is increasingly recognized as too narrow: The nature of the immune response to tumors also depends on innate immune cells such as macrophages and on the tumor micro- and macro-environment [85–88], which all impact T cells’ function and also play T cell–independent roles. Thus, from a clinical point of view, not only must one keep in mind that the concept of exhaustion takes different and often nonoverlapping meanings, but it is also essential to consider that immunological tolerance, be it mediated by “exhaustion” or not, is always dependent on “contextual” factors, some of which can be mechanistically studied and therapeutically manipulated.

**Fig 4:**
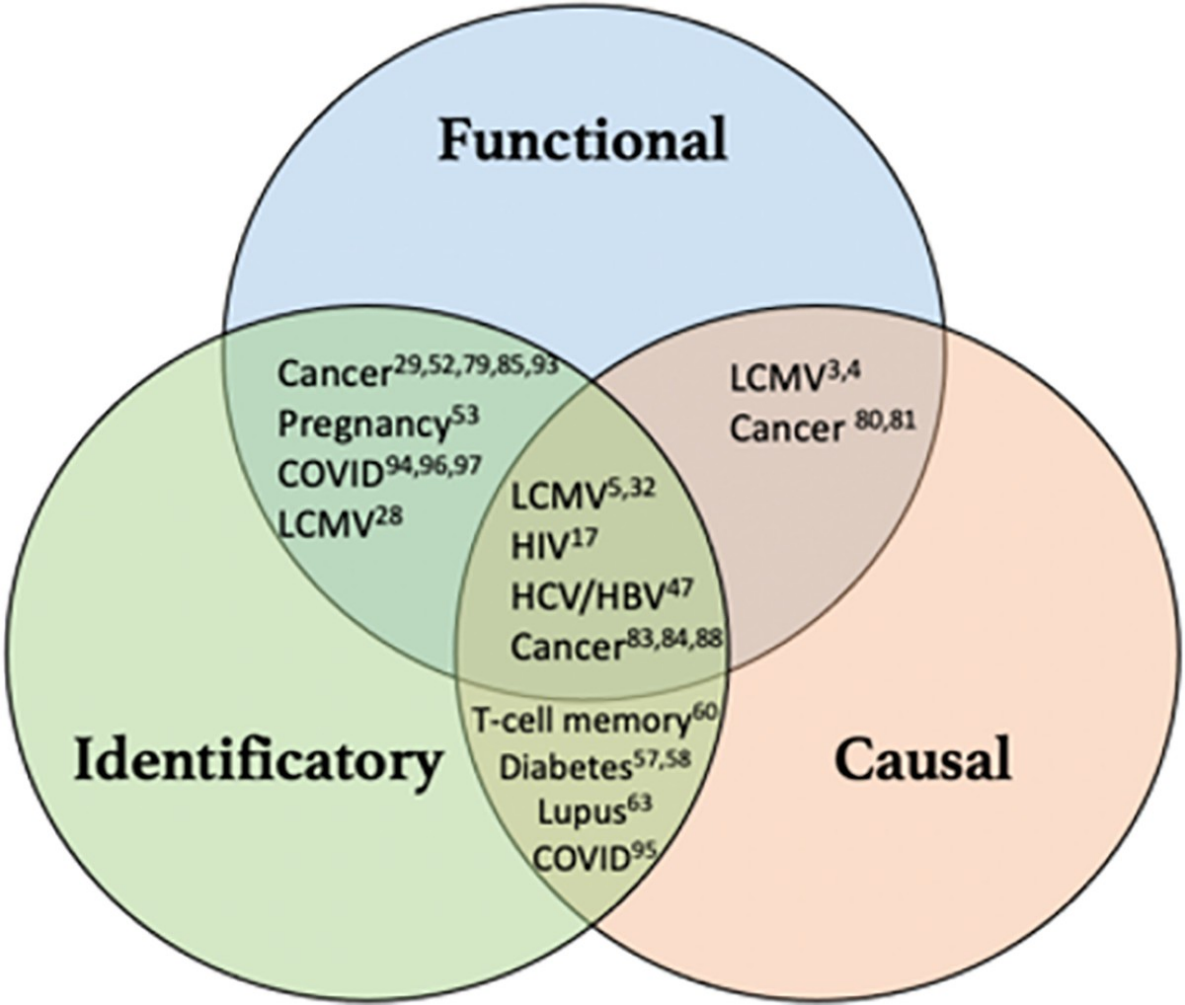
Examples of absence of overlap between the 3 approaches to exhaustion. COVID, Coronavirus Disease; HBV, hepatitis B virus; HCV, hepatitis C virus; LCMV, lymphocytic choriomeningitis virus.

Recent work on COVID-19 offers another interesting example of the potential inconsistencies and misunderstandings that can be associated with the concept of exhaustion. CD8+ T cells present an exhausted phenotype in many COVID-19 patients, but the cause involved is not always mentioned [89]. High antigen load seems to be a more likely cause than chronicity since the markers and the dysfunction of CD8+ T cells were observed in as short a period as 7 days postinfection [90,91]. Moreover, such T cells are not always described as dysfunctional [92]. Finally, it is now well known that COVID-19 patients can die from an overstimulation of the immune system, especially via “cytokine storms” [93]. Consequently, misunderstanding “the exhausted profile” as an indiscriminate need for T-cell reinvigoration could lead to clinical disaster for the patient. As with cancer, a productive application of the conceptual framework of exhaustion to COVID-19 will require a precise examination of the 3 approaches and a careful reflection on the full therapeutic consequences of the manipulation of “exhausted” T cells. **Fig 4** sums up examples of nonoverlap between the 3 approaches to exhaustion.

## Conclusions: Exploring new avenues about exhaustion and immunoregulation

From the 1990s to the present day, many immunologists have suggested that exhaustion might in fact reflect an evolutionary conserved process of immunoregulation [5,11], limiting the risk of immunopathology. Indeed, what immunologists have dubbed “exhaustion” to initially describe cells that seem to fail to realize their expected effector functions may well actually be one manifestation of a sometimes beneficial physiological process, preventing excessive immune responses and excessive damages.

One important way to approach this question is to adopt a multilevel perspective. A process that seems dysfunctional at a lower level can in fact be part of a functional regulatory process at a higher level. Typically, a state of exhaustion might be dysfunctional at the cell level while being functional at the tissue or organism level—for instance, if exhaustion helps limit immunopathology. This approach is convergent with a role for exhaustion in the phenomenon of “disease tolerance.” Disease tolerance, a term long used in plant ecology [94] and referring to a reduction of the negative impact of an infection on host fitness without directly affecting the pathogen burden, has been increasingly studied in recent immunology [95,96]. The phenomenon of disease tolerance reflects the fact that, in terms of fitness, it is sometimes better to mitigate the impact of a source of damage rather than eliminate it. Immunological exhaustion has indeed been characterized as one of the causes by which disease tolerance is achieved [97]. Future research will help clarify the concept of exhaustion thanks to a better understanding of its connections with both “disease tolerance” (which has to do with the non-elimination of a source of damage) and “immunological tolerance” (which has to do with the downregulation of an effector immune response).

The perspective presented in this paper opens up original and promising avenues for future research in at least 3 areas:

- **Regulation to self:** Future work needs to determine whether immune responses to “self” display some characteristics generally associated with exhaustion (in this case, people will presumably prefer to talk about “regulation”). Recent research has started to explore the role of exhaustion in the prevention of autoimmune diseases [26,98], but more work is needed, and the role of exhaustion must be investigated not only in autoimmune diseases but also in physiological autoimmunity (i.e., physiological responses to “self,” for example, in tissue maintenance and tissue repair). We expect that the experimental inhibition of exhaustion will favor autoimmune diseases and inflammatory diseases, as well as dysregulation of physiological responses to self-constituents.
- **Regulation to “resident nonself”:** Typically, the microbiota. Studies on the microbiota do not even examine the phenomenon of immunological exhaustion. An intriguing hypothesis would be that the experimental inhibition of exhaustion in the gut or on the skin might lead to elimination of some microbes with which the host normally cohabits.
- **Negative consequences of excessive limitation of exhaustion in contexts of the following:**
  (a) Infection: Although limiting exhaustion in some contexts of viral infection is a legitimate objective, we expect that an excessive inhibition of exhaustion could lead to pathological consequences, from immunopathology to the development of autoimmune disorders. The idea of an equilibrium between beneficial and detrimental exhaustion has been present for a long time in the literature on exhaustion and has been specifically investigated in recent times, but what is needed now is an explicit description of the causes that will insure an adequate equilibrium.
  (b) Cancer: There is no doubt that reversing T-cell exhaustion in some cancers can be useful. Yet, as we have seen, the sense given to the “exhaustion” of these cells is not consistently clear, and, in many cases, restoring the functions of these cells does not really amount to suppressing their state of “exhaustion.” Moreover, future research will certainly confirm that the “reinvigoration” of T cells in cancer may lead to detrimental consequences for the host in terms of immunopathology and autoimmune responses, which makes it all the more important to, first, specifically understand the type of immunoregulation (not necessarily reducible to exhaustion) involved in immune responses to cancer, and, second, systematically understand the causes of T-cell responses to cancer in the broader context of the many components of the immunological tumor microenvironment.
  (c) Allergies: Allergies are another domain in which, interestingly, exhaustion is almost never investigated. Future studies will have to determine if exhaustion plays a role in the allergic response, and, possibly, in desensitization (where the antigen is chronic but present in low quantities).

Discussing the definition of “exhaustion” is not just a matter of words. It has crucial consequences in experimental and clinical practice.
